# Obstetric characteristics and pregnancy outcomes under China's three-child policy: a retrospective cohort study

**DOI:** 10.3389/fgwh.2026.1781246

**Published:** 2026-05-04

**Authors:** Sisi Zhang, Weizhen Lin, Lihua Lin

**Affiliations:** 1 Department of Healthcare, Fujian Maternity and Child Health Hospital, Fuzhou, China; 2 Department of Medical, Fujian Maternity and Child Health Hospital, Fuzhou, China

**Keywords:** three-child policy, cesarean section, perinatal outcomes, maternal age, China

## Abstract

**Background:**

This retrospective cohort study aims to evaluate the association between maternal age, parity, and prior cesarean section (CS) with adverse perinatal outcomes under China's three-child policy and to assess whether the combination of advanced age, multiparity, and prior CS modifies these associations.

**Methods:**

Data were extracted from the monitoring information management system of 14 obstetric hospitals in Fujian Province between 1 January and 31 December 2023. A total of 27,002 deliveries were included. Maternal age was categorized as 20–34, 35–39, and ≥40 years. Parity was classified as primipara vs. multipara and prior CS as yes vs. no. Adjusted odds ratios (aOR) with 95% confidence intervals (CI) were calculated.

**Results:**

Among 27,002 women, advanced maternal age (≥40 years) alone was significantly associated with increased odds of gestational diabetes mellitus (GDM) (aOR = 1.98, 95% CI: 1.69–2.30) and hypertensive disorders of pregnancy (HDP) (aOR = 2.27, 95% CI: 1.65–3.11). The combination of advanced age, multiparity, and prior CS (three factors) was significantly associated only with anemia (aOR = 2.79, 95% CI: 2.39–3.27). No significant associations were observed for GDM, placenta previa, preterm birth, macrosomia, or low Apgar scores in the three-factor group. The lower odds of several outcomes (e.g., overall pregnancy complications and HDP) among multiparous women and those with prior cesarean section likely reflect selection bias.

**Conclusions:**

In the context of China's three-child policy, advanced maternal age remains a strong independent risk factor for GDM and HDP. However, the combination of advanced age, multiparity, and prior CS significantly increases only the risk of anemia. The apparent inverse associations for other outcomes are best explained by preferential childbearing among healthier women. Early identification of anemia and targeted interventions for high-risk pregnancies are essential for improving maternal and child health.

## 1 Background

Following the implementation of China's three-child policy in 2021, notable shifts in reproductive behavior have emerged, including a trend toward delayed childbearing and an increasing proportion of women of advanced maternal age (AMA) (1). Despite these demographic changes, national survey data indicate that fertility intentions for a third child remain modest, with only an estimated 4.5%–13% of respondents expressing willingness to have a third child—a figure that varies by age (2–5). AMA, commonly defined as maternal age 35 years or older at delivery, is a well-established independent risk factor for adverse pregnancy outcomes. Compared with younger women, women of AMA face elevated risks of miscarriage, preterm birth, gestational diabetes mellitus (GDM), preeclampsia, placenta previa, cesarean section (CS), and fetal chromosomal abnormalities (6–9). The prevalence of AMA pregnancies has been rising both globally and in China, accompanied by increases in medical comorbidities, use of assisted reproductive technology, and overall pregnancy complications. AMA pregnant women are more likely to experience miscarriage, preterm birth (both spontaneous and therapeutic), twin births, fetal chromosomal abnormalities, congenital structural anomalies, placenta previa, gestational diabetes mellitus, and preeclampsia, and advanced age is a significant contributing factor to cesarean section and pregnancy complications or adverse outcomes. However, according to data from different studies, although advanced age has been shown to be an independent risk factor, there is no conclusive evidence on the impact of large multiple pregnancies and advanced age at multiple births on adverse maternal outcomes.

However, despite the well-recognized risks associated with AMA, evidence remains limited regarding the combined effects of AMA, multiparity, and prior cesarean section on adverse perinatal outcomes in the context of China's updated fertility policy. In particular, the interactive effects of these factors—especially among women who are both of advanced age and have a history of cesarean delivery—have yet to be systematically examined in large-scale, multicenter studies in China since the policy change.

This study intends to present a retrospective multicenter study aimed to investigate the associations between maternal age, parity, prior cesarean section, and adverse perinatal outcomes under China's three-child policy and to assess whether the combination of these factors modifies the risk profile.

## 2 Methods

### 2.1 Definitions

We obtained the monitoring information management system for pregnant women's data for all newborns delivered in 14 hospitals. Our analysis was restricted to singleton births born alive with a gestational age at delivery ≥28 weeks. The gestational age in China is generally ascertained on the basis of the last menstrual period or ultrasound when the date of the last menstrual period is not known or when the menstrual cycle is irregular. The current gestational age is recorded in the maternal health booklet at each antenatal visit of a pregnant woman. Based on the delivery age of the pregnant woman, the parturients were divided into three groups: 20–34, 35–39, and over 40 years old. AMA has been defined to describe women who are 35 years or older on the estimated date of delivery. The number of antenatal care visits during pregnancy was categorized as 0, 1–4, 5–7, ≥8.

Maternal outcomes were classified into mutually exclusive categories as follows: direct obstetric complications: placenta previa, abruptio placentae, unspecified antepartum hemorrhage, and preeclampsia. Medical diseases included anemia (hemoglobin <110 g/L), and postpartum hemorrhage (PPH) was defined as blood loss exceeding 500 mL following vaginal delivery or 1,000 mL following cesarean section within 24 h after delivery (10).

Infant outcomes included four categories: preterm birth, low birth weight, macrosomia, and Apgar scores at 1 and 5 minutes (<7) (11, 12).

### 2.2 Data collection

**Figure 1:**
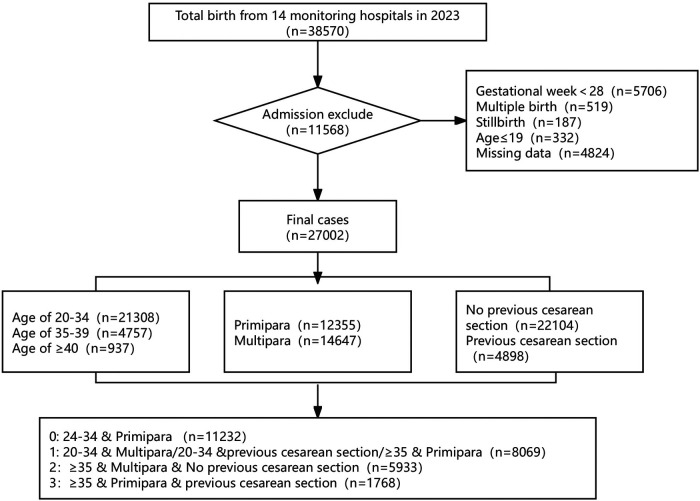
A flowchart of cases enrollment.

This retrospective study collected delivery data from the monitoring information management system for pregnant women across 14 hospitals in Fujian Province, China, between 1 January and 31 December 2023. A total of 38,570 deliveries were initially identified. Inclusion criteria were singleton live birth and gestational age ≥28 weeks. Exclusion criteria were maternal age <20 years, stillbirth, multiple gestations, and incomplete data. After applying these criteria, 27,002 deliveries were included in the final analysis. A detailed screening flowchart is provided in Figure 1. Written informed consent was obtained from all participants, and all procedures were performed in accordance with relevant regulations.

### 2.3 Statistical analysis

Categorical variables were presented as frequencies (percentages). Data were cleaned for errors prior to data analysis. Inconsistencies and missing values were checked visually using frequency distributions. A multivariable logistic regression analysis was performed to predict each pregnancy outcome. The covariates included in the multivariable logistic regression models were maternal education level, marital status, gravidity, previous cesarean section, age, mode of delivery, hospital level, antenatal care, parity, and fetal sex. The selection of these covariates was based on prior literature and clinical relevance. The outcomes assessed included both maternal outcomes and infant outcomes. All statistical analyses were conducted using Excel and SPSS version 21 and R version 4.5.3. Statistically significant associated factors were identified based on a two-tailed *p*-value ≤0.05. To account for hospital-level clustering in this multicenter study, we fitted mixed-effects logistic regression models with a random intercept for each hospital. The between-hospital variance was 0.0347, and the intraclass correlation coefficient was approximately 1.04% [0.0347/(0.0347 + π²/3)], indicating small but non-negligible heterogeneity across hospitals. The model AIC was 11,915.5. Residual diagnostics revealed a minimum residual of −11.09; however, the binomial logistic model is robust to such deviations, and the reported adjusted odds ratios (aORs) and 95% confidence intervals (CIs) are considered reliable.

## 3 Results

### 3.1 Obstetric characteristics of women under China's “three-child policy”: a descriptive analysis

Table 1 presents the maternal characteristics and delivery details of the 27,002 deliveries included in the study. The majority of women were aged 20–34 years, had a high level of education (64.75% [17,483/27,002]), were married (98.71% [26,653/27,002]), were multiparous (54.24% [12,355/27,002]), and had received at least eight antenatal visits (88.76% [23,966/27,002]). A substantial proportion of deliveries took place in provincial hospitals (43.42% [11,724/27,002]).

**Table 1:** Maternal characteristics and delivery information.

Variables	Frequency	Percent	Variables	Frequency	Percent
Age			Mode of delivery		
20–34	21,308	78.91	Vaginal delivery	14,932	55.30
35–39	4,757	17.62	Cesarean section	12,070	44.70
≥40	937	3.47	Parity		
Maternal education degree			0	12,355	45.76
College or above	17,483	64.75	1	11,229	41.59
High school	4,164	15.42	≥2	3,418	12.66
Middle school	4,694	17.38	Fetal sex		
Primary school or below	661	2.45	Male	14,093	52.19
Marital status			Female	12,907	47.80
Married	26,653	98.71	Unknown	2	0.01
Single, widowed, or divorced	349	1.29			
Gravidity			Maternal		
Primipara	12,355	45.76	Pregnancy complications	21,867	80.98
Multipara	14,647	54.24	Anemia	5,289	19.59
Previous CS			GDM	6,288	23.29
No previous cesarean section	22,104	81.86	HDP	1,228	4.55
Previous cesarean section	4,898	18.14	PPH	6,076	22.5
Antenatal care			PE	508	1.88
0	125	0.46	Placenta previa	259	0.96
1–4	926	3.43	Placental abruption	344	1.27
5–7	1,985	7.35	Infant		
≥8	23,966	88.76	Preterm	1,704	6.31
Hospital level			LBW	1,287	4.77
Provincial hospital	11,724	43.42	Macrosomia	878	3.25
Municipal hospital	8,020	29.70	Apgar score at 1 min <7	104	0.39
County hospital	7,258	26.88	Apgar score at 5 min <7	41	0.15

With regard to maternal outcomes, the overall proportion of pregnancy complications was 80.98% (21,867/27,002), which is higher than previously reported rates in Hebei Province, China (10). GDM was 23.29% (6,288/27,002), anemia 19.59% (5,289/27,002), and cesarean delivery 44.70% (12,070/27,002).

For infant outcomes, the proportion of preterm birth was 6.31% (1,704/27,002), low birth weight (LBW) 4.77% (1,287/27,002), and macrosomia 3.25% (878/27,002). These findings highlight a high burden of maternal and infant complications in this population, underscoring the need for targeted interventions.

### 3.2 Multivariable analysis of adverse outcomes by advanced maternal age

Table 2 shows that advanced maternal age (≥35 years) was associated with increased odds of several adverse outcomes in a dose-dependent manner. Compared with women aged 20–34 years, those aged 35–39 years had higher odds of GDM (aOR = 1.53, 95% CI: 1.42–1.64), HDP (aOR = 1.43, 95% CI: 1.18–1.74), and PPH (aOR = 1.12, 95% CI: 1.02–1.23). Women aged ≥40 years showed even greater odds for GDM (aOR = 1.98, 95% CI: 1.69–2.30) and HDP (aOR = 2.27, 95% CI: 1.65–3.11). No significant associations were observed for infant outcomes across age groups.

**Table 2:** Adverse outcomes of individuals of different ages.

Variables	20–34 (*n* = 21,308)		35–39 (*n* = 4,757)		≥40 (*n* = 937)	
	*n* (%)	aOR (95% CI)	*n* (%)	aOR (95% CI)	*n* (%)	aOR (95% CI)
Maternal						
Pregnancy complications	17,101 (80.26)	1	3,952 (83.08)	1.05 (0.95,1.16)	814 (86.87)	1.18 (0.94,1.48)
Anemia	3,950 (18.54)	1	1,072 (22.54)	1.00 (0.92,1.09)	267 (28.50)	1.14 (0.96,1.34)
GDM	4,516 (21.19)	1	1,432 (30.10)	1.53 (1.42,1.64)	340 (36.29)	1.98 (1.69,2.30)
HDP	881 (4.13)	1	263 (5.53)	1.43 (1.18,1.74)	84 (8.96)	2.27 (1.65,3.11)
PPH	4,865 (22.83)	1	1,028 (21.61)	1.12 (1.02,1.23)	183 (19.53)	1.22 (0.99,1.49)
PE	370 (1.74)	1	105 (2.21)	0.84 (0.62,1.14)	33 (3.52)	0.77 (0.47,1.26)
Placenta previa	163 (0.76)	1	76 (1.60)	1.31 (0.97,1.77)	20 (2.13)	1.25 (0.75,2.08)
Placental abruption	257 (1.21)	1	70 (1.47)	1.21 (0.92,1.61)	17 (1.81)	1.49 (0.88,2.52)
Infant						
Preterm	1,261 (5.92)	1	357 (7.50)	0.94 (0.79,1.11)	86 (9.18)	0.89 (0.65,1.21)
LBW	962 (4.51)	1	262 (5.51)	1.11 (0.92,1.34)	63 (6.72)	1.09 (0.77,1.57)
Macrosomia	641 (3.01)	1	19 (4.14)	1.12 (0.94,1.34)	40 (4.27)	1.06 (0.76,1.49)
Apgar score at 1 min<7	79 (0.37)	1	16 (0.34)	0.91 (0.49, 1.68)	9 (0.96)	1.59 (0.67,3.79)
Apgar score at 5 min<7	34 (0.16)	1	4 (0.08)	0.55 (0.17,1.74)	3 (0.32)	1.28 (0.30,5.43)

Nagelkerke R² = 0.172. aOR, adjusted odds ratio; 95% CI, 95% confidence interval. Outcomes coded as 0 = no event, 1 = event. Adjusted for marital status, maternal education, gravidity, mode of delivery, antenatal care, hospital level, parity, and fetal sex.

### 3.3 Multivariable analysis of adverse outcomes by parity

Table 3 presents aOR with CI for adverse maternal and infant outcomes, comparing multiparous women (*n* = 14,647) to primiparous women (*n* = 12,355; reference). No covariate adjustment was performed.

**Table 3:** Adverse outcomes of individuals of different gravidity.

Variables	Primipara (*n* = 12,355)		Multipara (*n* = 14,647)	
	*n* (%)	aOR (95% CI)	*n* (%)	aOR (95% CI)
Maternal				
Pregnancy complications	10,199 (2.55)	1	11,668 (79.66)	0.70 (0.65, 0.76)
Anemia	2,106 (17.05)	1	3,183 (21.73)	1.22 (1.14,1.31)
GDM	2,728 (22.08)	1	3,560 (24.31)	0.97 (0.91,1.04)
HDP	674 (5.46)	1	554 (3.78)	0.54 (0.46,0.65)
PPH	2,862 (23.16)	1	3,214 (21.94)	1.25 (1.17, 1.34)
PE	287 (2.30)	1	221 (1.50)	0.94 (0.72, 1.23)
Placenta previa	83 (0.67)	1	176 (1.20)	1.16 (0.86, 1.56)
Placental abruption	167 (1.35)	1	177 (1.21)	1.00 (0.79, 1.27)
Infant				
Preterm	707 (5.72)	1	997 (6.81)	1.22 (1.06, 1.41)
LBW	608 (4.92)	1	679 (4.64)	0.77 (0.66,0.91)
Macrosomia	338 (2.74)	1	540 (3.69)	1.15 (0.98, 1.34)
Apgar score at 1 min<7	48 (0.39)	1	56 (0.38)	0.86 (0.52, 1.43)
Apgar score at 5 min<7	23 (0.19)	1	18 (0.12)	0.57 (0.26, 1.25)

Nagelkerke R² = 0.285. aOR, adjusted odds ratio; 95% CI, 95% confidence interval. Outcomes coded as 0 = no event, 1 = event. Adjusted for maternal age, education, marital status, antenatal care, hospital level, and fetal sex.

Multiparous women had significantly lower odds of overall pregnancy complications (aOR = 0.70, 95%  CI: 0.65–0.76) and hypertensive disorders of pregnancy (HDP) (aOR = 0.54, 95%  CI: 0.46–0.65). In contrast, multiparity was associated with higher odds of anemia (aOR = 1.22, 95% CI: 1.14–1.31), PPH (aOR = 1.25, 95%  CI: 1.17–1.34), and preterm birth (aOR = 1.22, 95%  CI: 1.06–1.41). No statistically significant differences were observed for gestational diabetes mellitus (GDM), preeclampsia, placenta previa, placental abruption, macrosomia, or low Apgar scores at 1 or 5 min.

### 3.4 Multivariable analysis of adverse outcomes by previous cesarean section

Table 4 shows adjusted ORs comparing women with a previous cesarean section (*n* = 4,898) with those without (*n* = 22,104; reference). Women with a prior cesarean section had significantly higher odds of anemia (aOR = 2.33, 95%  CI: 2.13–2.54) and GDM (aOR = 1.19, 95%  CI: 1.09–1.29) and significantly lower odds of overall pregnancy complications (aOR = 0.87, 95%  CI: 0.79–0.97), PPH (aOR = 0.16, 95%  CI: 0.14–0.18), and placental abruption (aOR = 0.37, 95%  CI: 0.25–0.57). No significant associations were detected for HDP, preeclampsia, placenta previa, preterm birth, LBW, macrosomia, or low Apgar scores.

**Table 4:** Adverse outcomes of individuals with a previous cesarean section.

Variables	No previous cesarean section (*n* = 22,104)		previous cesarean section (*n* = 4,898)	
	*n* (%)	aOR (95% CI)	*n* (%)	aOR (95% CI)
Maternal				
Pregnancy complications	18,016 (81.51)	1	3,851 (78.62)	0.87 (0.79,0.97)
Anemia	3,720 (16.83)	1	1,569 (32.03)	2.33 (2.13,2.54)
GDM	4,971 (22.49)	1	1,317 (26.89)	1.19 (1.09,1.29)
HDP	1,009 (4.56)	1	219 (4.47)	1.04 (0.83,1.31)
PPH	5,743 (25.98)	1	333 (6.80)	0.16 (0.14,0.18)
PE	417 (1.89)	1	91 (1.86)	0.92 (0.65,1.29)
Placenta previa	178 (0.81)	1	81 (1.65)	1.13 (0.83,1.54)
Placental abruption	313 (1.42)	1	31 (0.63)	0.37 (0.25,0.57)
Infant				
Preterm	1,306 (5.91)	1	398 (8.13)	1.13 (0.95,1.35)
LBW	1,025 (4.64)	1	262 (5.35)	0.91 (0.74,1.11)
Macrosomia	692 (3.13)	1	186 (3.80)	0.92 (0.76,1.11)
Apgar score at 1 min<7	78 (0.35)	1	26 (0.53)	1.58 (0.88,2.83)
Apgar score at 5 min<7	34 (0.15)	1	7 (0.14)	0.58 (0.21,1.64)

Nagelkerke R² = 0.327. aOR, adjusted odds ratio; 95% CI, 95% confidence interval. Outcomes coded as 0 = no event, 1 = event. Adjusted for maternal age, education, marital status, antenatal care, hospital level, and fetal sex.

### 3.5 Combined effects of advanced maternal age, multiparity, and prior cesarean section

Table 5 examines the cumulative impact of three risk factors—AMA, multiparity, and previous cesarean section—on perinatal outcomes using adjusted ORs. The reference group comprised women with none of these factors (*n* = 11,232). Groups with one (*n* = 8,069), two (*n* = 5,933), and three (*n* = 1,768) factors were compared.

**Table 5:** Adverse perinatal outcomes associated with the combined presence of three risk factors.

Variables	0 (*n* = 11,232)		1 (*n* = 8,069)		2 (*n* = 5,933)		3 (*n* = 1,768)	
	*n* (%)	aOR (95% CI)	*n* (%)	aOR (95% CI)	*n* (%)	aOR (95% CI)	*n* (%)	aOR (95% CI)
Maternal								
Pregnancy complications	9,208 (81.98)	1	6,451 (79.95)	0.76 (0.69,0.83)	4,790 (80.73)	0.67 (0.60,0.76)	1,418 (80.20)	0.51 (0.43,0.62)
Anemia	1,880 (16.74)	1	1,305 (16.17)	0.96 (0.88,1.05)	1,526 (25.72)	1.81 (1.64,2.00)	578 (32.69)	2.79 (2.39,3.27)
GDM	2,335 (20.79)	1	1,819 (22.54)	0.97 (0.90,1.06)	1,572 (26.50)	1.03 (0.94,1.14)	562 (31.79)	1.13 (0.97,1.32)
HDP	10,662 (94.93)	1	7,767 (96.26)	0.64 (0.52,0.79)	5,683 (95.79)	0.56 (0.43,0.71)	1,662 (94.00)	0.55 (0.37,0.79)
PPH	8,577 (76.36)	1	5,863 (72.66)	1.45 (1.33,1.57)	4,840 (81.58)	0.79 (0.71,0.87)	1,646 (93.10)	0.19 (0.16,0.25)
PE	244 (2.17)	1	122 (1.51)	0.97 (0.71,1.34)	98 (1.65)	0.84 (0.57,1.25)	44 (2.49)	0.86 (0.49,1.52)
Placenta previa	67 (0.60)	1	68 (0.84)	1.07 (0.74,1.55)	87 (1.47)	1.18 (0.79,1.75)	37 (2.09)	1.26 (0.74,2.14)
Placenta abruption	148 (1.32)	1	111 (1.38)	1.16 (0.88.1.52)	71 (1.20)	0.84 (0.59,1.19)	14 (0.79)	0.42 (0.22,0.82)
Infant								
Preterm	621 (5.53)	1	486 (6.02)	1.15 (0.97,1.36)	439 (7.40)	1.27 (1.04,1.55)	158 (8.94)	1.33 (0.98,1.78)
LBW	531 (4.73)	1	353 (4.37)	0.83 (0.69,1.00)	296 (4.99)	0.76 (0.60,0.95)	107 (6.05)	0.70 (0.49,0.99)
Macrosomia	300 (2.67)	1	280 (3.47)	1.16 (0.97,1.39)	211 (3.56)	1.03 (0.83,1.28)	87 (4.92)	1.28 (0.93,1.76)
Apgar score at 1 min<7	42 (0.37)	1	30 (0.37)	0.92 (0.50,1.67)	19 (0.32)	0.72 (0.34,1.51)	13 (0.74)	1.81 (0.70,4.64)
Apgar score at 5 min<7	20 (0.18)	1	12 (0.15)	0.73 (0.29,1.77)	7 (0.12)	0.63 (0.20,1.93)	2 (0.11)	0.19 (0.02,1.57)

Nagelkerke R² = 0.471. aOR, adjusted odds ratio; 95% CI, 95% confidence interval. Outcomes coded as 0 = no event, 1 = event. Adjusted for maternal age, education, marital status, antenatal care, hospital level, and fetal sex.

Compared with the reference group, women with all three factors demonstrated progressively higher odds of anemia (aOR = 2.79, 95%  CI: 2.39–3.27), GDM (aOR = 1.13, 95%  CI: 0.97–1.32, not significant), placenta previa (aOR = 1.26, 95%  CI: 0.74–2.14, not significant), preterm birth (aOR = 1.33, 95%  CI: 0.98–1.78, not significant), macrosomia (aOR  = 1.28, 95%  CI: 0.93–1.76, not significant), and low 1 min Apgar score (aOR = 1.81, 95%  CI: 0.70–4.64, not significant). Conversely, women with three factors had significantly lower odds of overall pregnancy complications (aOR = 0.51, 95%  CI: 0.43–0.62), HDP (aOR = 0.55, 95%  CI: 0.37–0.79), PPH (aOR = 0.19, 95%  CI: 0.16–0.25), placental abruption (aOR = 0.42, 95%  CI: 0.22–0.82), and LBW (aOR = 0.70, 95%  CI: 0.49–0.99).

## 4 Discussion

Under China's three-child policy, women who choose to have a third child often present with AMA, multiparity, and a history of previous CS—each independently confirmed as risk factors for adverse perinatal outcomes (13–16). This study examined the combined effects of these three characteristics within the policy context. The principal finding is that the simultaneous presence of AMA, multiparity, and prior CS is associated with a broader spectrum of adverse outcomes compared with any single factor alone, although many of the associations for the three-factor combination did not reach statistical significance.

Specifically, compared with women with none of the three risk factors, those with all three factors had significantly higher odds of anemia (aOR = 2.79, 95%  CI: 2.39–3.27) (Table 5). No significant increases were observed for GDM (aOR = 1.13, 95%  CI: 0.97–1.32), placenta previa (aOR = 1.26, 95%  CI: 0.74–2.14), preterm birth (OR = 1.33, 95%  CI: 0.98–1.78), macrosomia (aOR = 1.28, 95%  CI: 0.93–1.76), or low 1 min Apgar score (aOR = 1.81, 95%  CI: 0.70–4.64). In contrast, the three-factor combination was associated with significantly lower odds of overall pregnancy complications (aOR = 0.51, 95%  CI: 0.43–0.62), HDP (aOR = 0.55, 95%  CI: 0.37–0.79), PPH (aOR = 0.19, 95%  CI: 0.16–0.25), placental abruption (aOR = 0.42, 95%  CI: 0.22–0.82), and LBW (aOR = 0.70, 95%  CI: 0.49–0.99).

The rate of prevalence of anemia among women with all three factors was 32.69%, which exceeds that reported in Japan (23.4%) and in Europe/North America (11.5%–19.2%) (15). The preterm birth rate in this subgroup (8.94%) was slightly above the 2020 national average for China (6.1%) (17). The overall GDM prevalence in our cohort (31.79% among women with all three factors) was substantially higher than the pooled estimate from 25 Chinese studies (14.8%) (18, 19). Notably, advanced maternal age alone (≥40 years) showed a stronger association with GDM (aOR = 1.98, 95%  CI: 1.69–2.30) and HDP (aOR = 2.27, 95%  CI: 1.65–3.11) than did the three-factor combination (Table 2), suggesting that age is the dominant driver for these conditions.

The inverse associations observed for pregnancy complications, HDP, PPH, placental abruption, and LBW among women with the three-factor combination should not be interpreted as protective biological effects. Rather, they most likely reflect selection bias: women who experience severe complications (e.g., HDP, severe PPH) in earlier pregnancies are less likely to proceed to subsequent pregnancies, making the group with multiple risk factors a healthier subset of the population. Similar inverse associations have been reported elsewhere and are generally attributed to the same bias (20).

Consistent with previous work (7, 21, 22), we observed that AMA was associated with a higher incidence of several adverse outcomes compared with younger age (Table 2). Among multiparous women, AMA increased the risks of GDM, HDP, and PPH (for age 35–39 years: GDM aOR = 1.53, HDP aOR = 1.43, PPH aOR = 1.12). However, when the three factors (AMA, multiparity, and prior CS) were considered jointly, the independent contributions of multiparity and prior CS did not further elevate risks for GDM, preterm birth, or macrosomia beyond the effect of AMA alone. This suggests that the cumulative risk model may be more complex than a simple additive effect, and future studies with larger sample sizes are needed to clarify potential interactions.

In summary, the combination of AMA, multiparity, and prior CS is associated with substantially higher odds of anemia but not with other adverse outcomes after accounting for selection bias. The inverse associations for other complications likely reflect that healthier women are more likely to have subsequent pregnancies.

## 5 Conclusions

Following the shift in China's birth policy from the one-child policy to the universal two-child policy, Zhang et al. also reported an increased proportion of women of advanced reproductive age, who tended to have higher education levels and greater multiparity (23). In the present study, advanced maternal age (≥35 years) was independently associated with increased odds of GDM and HDP, with the highest risks observed in women aged ≥40 years (GDM: aOR = 1.98; HDP: aOR = 2.27). However, the combination of advanced maternal age, multiparity, and a previous cesarean section was significantly associated only with anemia (aOR = 2.79, 95% CI: 2.39–3.27) and did not significantly increase the odds of GDM, preterm birth, macrosomia, or low Apgar scores after accounting for selection bias. The inverse associations for overall pregnancy complications, HDP, postpartum hemorrhage, placental abruption, and low birth weight in the three-factor group likely reflect selection bias, as healthier women with uncomplicated prior pregnancies are more likely to have subsequent children.

The timing of delivery should be individualized based on maternal and fetal factors, and routine postponement of delivery beyond the late weeks of pregnancy is not recommended. While advanced maternal age is associated with an increased risk of obstructed labor and cesarean section, age alone is not an indication for cesarean delivery. In the absence of other maternal or fetal indications, vaginal delivery should be encouraged for older pregnant women. For women with a prior cesarean section who are undergoing a third pregnancy, special attention is warranted, and adherence to cesarean section indications becomes even more critical. Antenatal screening is particularly crucial in this group. To effectively support the three-child policy, comprehensive policy protections for pregnant women are essential. This includes heightened screening for gestational diabetes mellitus and anemia among women with combined risk factors—with particular emphasis on anemia, which showed the strongest association with the triple-risk combination in this study—thorough counseling on the risks associated with multiple prior cesarean sections, and expansion of the maternal and child health workforce to provide professional medical guidance and help prevent adverse outcomes.

## 6 Limitations

This study has several limitations. First, selection bias may be present in the multiparous group, as women with prior pregnancy complications are less likely to have subsequent children, thereby potentially biasing the observed associations. Second, the study period was limited to a single year (2023), and the sample included relatively few women with three or more deliveries, which limits the generalizability of the findings. Third, despite adjusting for multiple covariates in the multivariable analysis, residual confounding due to unmeasured factors—such as socioeconomic status, lifestyle, and antenatal care—cannot be ruled out given the retrospective design. A prospective cohort study is warranted to further ascertain obstetric safety and identify causal risk factors. In future research, we aim to address these limitations by enhancing sample representativeness, extending the study period, and more precisely delineating the determinants of adverse pregnancy outcomes under China's three-child policy.

## Data Availability

The datasets presented in this article are not readily available because Not applicable. Requests to access the datasets should be directed to Sisi Zhang, shissya@163.com.
